# A Study of C2C12 Myoblast Bioenergetics in Response to the CCG-1423 Rho A Inhibitor

**DOI:** 10.1093/geroni/igab046.2567

**Published:** 2021-12-17

**Authors:** Bachkhoa Nguyen, Fathima Ameer, Jasmine Crane, Gohar Azhar, Xiaomin Zhang, Jeanne Wei

**Affiliations:** 1 University of Arkansas for Medical Sciences, Little Rock, Arkansas, United States; 2 UAMS, UAMS, Arkansas, United States

## Abstract

CCG-1423 is a Rho A pathway inhibitor which has been reported to inhibit Rho/SRF-mediated transcriptional regulation. SRF and SRF cofactors, which include ternary complex factors (TCFs) and myocardin-related transcription factor (MRTF), regulate various cellular functions. The Rho/SRF signaling pathway also regulates the sirtuin 2 (SIRT2) gene that contains a classic serum response element (SRE) sequence. Current research on CCG-1423 focuses on gene expression levels of SRF in response to CCG-1423 and how SRF levels affect the cells; the studies are focused on cell morphology, migration, viability/reproduction, and overall function. The pathways of this inhibitor have yet to be fully elucidated, but several have been suggested with good evidence. Our goal is to study the effect of CCG-1423 on mitochondrial function and gene expression of cells. In this work C2C12 myoblast cells have been used as an in-vitro model to study cellular bioenergetics and variations in gene expressions induced by CCG-1423. The effect of CCG-1423 on mitochondrial function was determined by measuring the mitochondrial oxygen consumption rate and glycolysis rate after treating C2C12 cells with varying concentrations of CCG-1423 overnight. In C2C12 myoblast cells, CCG-1423 treatment significantly reduced mitochondrial oxygen consumption rate (OCR) in a dose-dependent manner. However, treatment of C2C12 cells with CCG-1423 overnight increased the extracellular acidification rate (ECAR) in a dose-dependent manner. By indicating that CCG-1423 represses mitochondrial respiration via the Rho-SRF signaling pathway, the results of this study may enable a better understanding of the bioenergetics of the cell in the aging body.

